# Tougher clots, safer lives? Revisiting the role of factor XIII in thrombus stability and embolization risk

**DOI:** 10.1016/j.rpth.2025.102901

**Published:** 2025-05-23

**Authors:** Zsuzsa Bagoly

**Affiliations:** 1 Division of Clinical Laboratory Science, Department of Laboratory Medicine, Faculty of Medicine, University of Debrecen, Debrecen, Hungary; 2 Hungarian Academy of Sciences (MTA-DE) Lendület “Momentum” Hemostasis and Stroke Research Group, Debrecen, Hungary; 3 Hungarian Research Network (HUN-REN-DE) Cerebrovascular Research Group, Debrecen, Hungary

Thromboembolism remains a major contributor to global morbidity and mortality, with embolic events such as pulmonary embolism and ischemic stroke accounting for substantial acute and long-term clinical burden [1]. While the coagulation cascade has been exhaustively studied from the perspective of clot formation, less attention has been given to clot vulnerability—specifically, the mechanical rupture of thrombi that leads to embolization [2]. In their recent article, Ramanujam et al. [3] provide compelling mechanistic data linking the crosslinking activity of activated factor (F)XIII (FXIIIa) to the physical integrity of fibrin clots, particularly their resistance to rupture under stress. This study offers a timely and sophisticated contribution to our understanding of clot biomechanics, with potential ramifications for both diagnostic stratification and therapeutic modulation in thromboembolic disease.

One of the most innovative aspects of this study and other recent studies of this group is its application of fracture mechanics—a domain more often associated with materials science than hematology—to characterize fibrin clot stability [2,3]. Specifically, the authors quantify fracture toughness (Gc), which describes a material’s ability to resist crack propagation. This is particularly relevant to thrombosis, where clot heterogeneity and mechanical stress from flowing blood can trigger failure in structurally vulnerable regions.

The mechanistic decoupling—between structure and stability—has important implications. FXIIIa-mediated covalent bonds between fibrin chains enhance the collective ability of the network to distribute and absorb stress, preventing localized strain concentrations that precipitate rupture. Using iodoacetamide to selectively inhibit FXIIIa-mediated fibrin crosslinking, Ramanujam et al. [3] demonstrated a striking dose-dependent reduction in fracture toughness, from 8.6 to 2.3 N/m, with minimal changes in extensibility [3]. This not only aligns with previous single-fiber studies demonstrating stiffer fibers following full FXIIIa crosslinking compared with uncrosslinked fibers [4] but also extends the paradigm to whole fibrin clot mechanics under biologically relevant strain conditions. Rheological and microscopy data further confirmed reduced fibrin stiffness and minor alterations in fiber architecture. Notably, these findings suggest that clot stability is not solely determined by gross morphologic features such as fiber thickness or density, but by the cooperative interactions enabled by FXIIIa crosslinks. This concept has been supported by in vivo data by Duval et al. [5], demonstrating that genetic elimination of fibrin γ-chain crosslinking in mice resulted in significantly increased pulmonary embolism despite normal clot size and fibrinolysis, highlighting the critical role of γ-chain crosslinking in maintaining thrombus integrity and preventing embolization [5].

The translational potential of these findings is considerable. As the authors rightly highlight, clots formed under FXIIIa-deficient conditions—even though they appear morphologically intact—exhibit markedly lower resistance to rupture (Figure). Therefore, one should not think of embolization as merely a function of clot size or composition, but of mechanical fragility in the context of hemodynamic forces. This has direct relevance to a spectrum of clinical settings.

**Figure:**
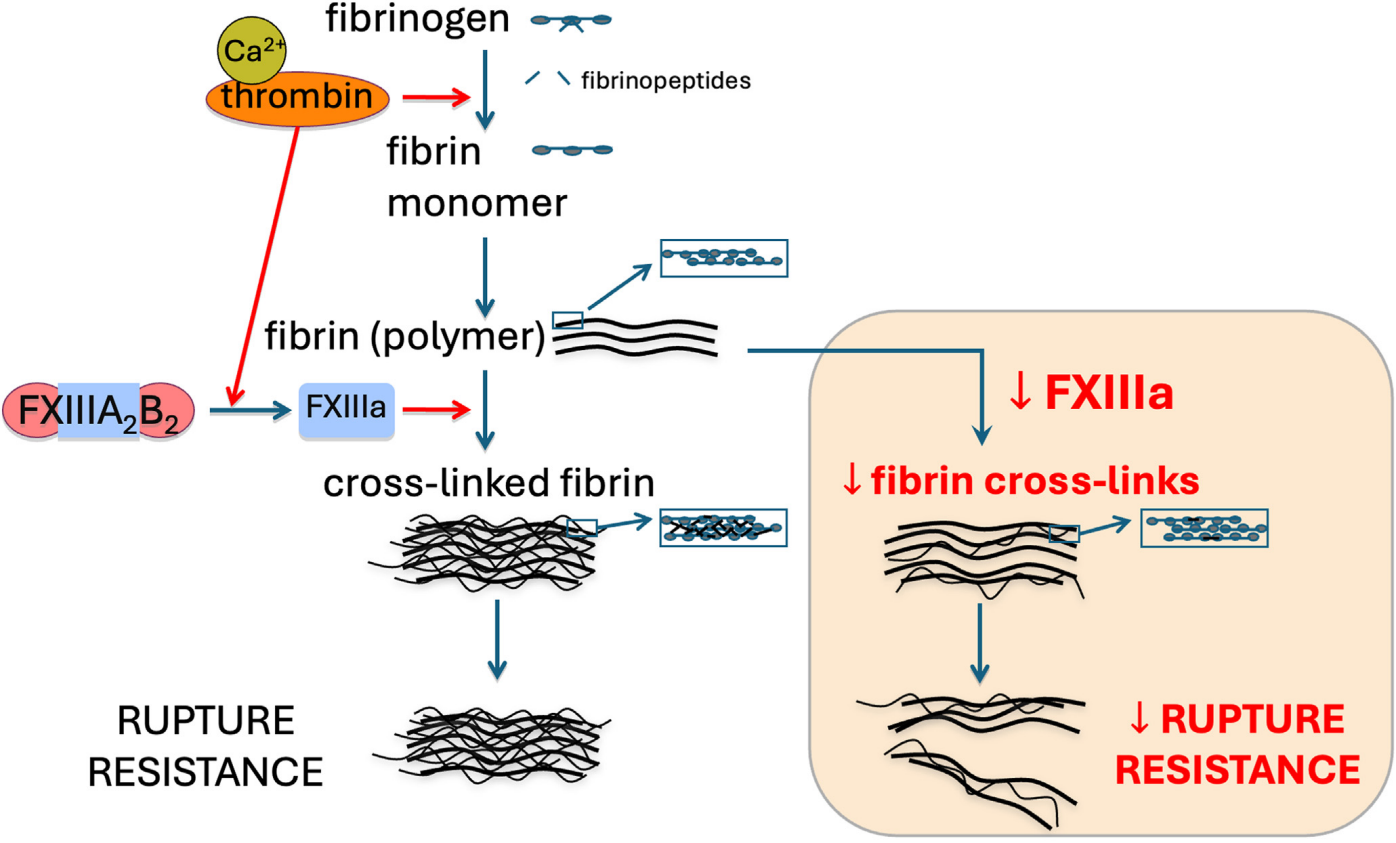
The central role of activated factor (F)XIII (FXIIIa) in clot stability and rupture resistance. Schematic overview illustrating that FXIIIa-mediated crosslinking strengthens fibrin networks via increased mechanical cooperativity, thereby enhancing fracture toughness and reducing the likelihood of clot rupture and embolization. In clinical scenarios when FXIII levels are reduced (beige panel), decreased fibrin crosslinking leads to decreased resistance against clot rupture and increased likelihood of embolization.

Although congenital FXIII deficiency is a relatively rare condition, acquired FXIII deficiency (immune mediated or nonimmune mediated, such as in liver disease, sepsis, COVID-19, or certain hematologic malignancies, etc) is more frequent [[6], [7], [8]]. In these cases, patients may exhibit clot instability, which will not necessarily be detected by conventional laboratory methods. FXIII levels can be reduced in acute thrombotic events, particularly in pulmonary embolism [9], and the study by Ramanujam et al. [3] provides a plausible mechanistic link between such reductions and increased embolic risk. These findings might be highly relevant in certain specific clinical scenarios or treatments, eg, during cardiopulmonary bypass or extracorporeal membrane oxygenation treatment when repetitive mechanical strain is imposed on newly formed thrombi, making FXIII levels a potentially under recognized determinant of clot integrity and embolic complications in these contexts [10].

From a therapeutic perspective, augmenting FXIII levels (eg, with concentrates or recombinant form) could be reconsidered not only to prevent bleeding but also to enhance thrombus stability and reduce embolic risk. Although thrombotic events in FXIII-deficient patients are a rarity, the management of anticoagulation and FXIII replacement in such clinical situations are highly challenging [11]. Conversely, reducing the levels or targeting activation or activity of FXIIIa might be explored as a strategy to destabilize pathologic thrombi in selected clinical cases, although such approaches would require careful balance to avoid hemorrhagic complications [7].

Despite the robust in vitro methods used by Ramanujam et al. [3], several questions remain. First, translating mechanical fracture properties to in vivo thrombosis models is essential. Blood flow, cellular components (eg, platelets, red blood cells, neutrophils, and neutrophil extracellular traps), and variable thrombus composition introduce complexities that may modulate the contribution of FXIIIa to clot behavior [12,13]. For example, previous studies using a venous thromboembolism model in mice that permits embolization of red blood cell-rich and fibrin-rich thrombi lead to an observation that only complete FXIII deficiency increases pulmonary embolism incidence, while partial FXIII deficiency does not [13].

Second, the contribution of the fibrinolytic system to embolization risk must not be underestimated. FXIIIa not only stabilizes fibrin clots by crosslinking fibrin strands but also incorporates antifibrinolytic proteins such as α2-plasmin inhibitor into the newly formed fibrin clot in a concentration-dependent manner, thereby making the clot more resistant to plasmin-mediated degradation [14,15]. The tightly regulated process of fibrinolysis represents a critical determinant of thrombus stability, while dysregulated fibrinolysis—whether excessive or insufficient—can alter embolization risk. Hyperfibrinolysis may undermine clot mechanical integrity [16], especially in the context of reduced FXIIIa-mediated crosslinking of fibrin strands and α2-plasmin inhibitor, rendering thrombi more susceptible to lysis and fragmentation under shear stress. Conversely, hypofibrinolytic states may promote thrombus propagation and prolong intravascular persistence, creating a reservoir for delayed embolic events [17].

Third, individual variability in clot mechanics and lysis susceptibility is most likely influenced by common genetic polymorphisms in FXIII (eg, FXIII-A Val34Leu) or in fibrinogen, as well as by fibrinogen levels, which may significantly alter crosslinking kinetics or fibrin architecture [18,19]. It would be highly interesting to see future studies examining these variants in patient-derived plasma samples and integrating genome-wide association data with biomechanical profiling.

Finally, the development of diagnostic tools that reflect clot toughness—rather than just clot formation or lysis time—is an exciting but unmet challenge [20]. Potential approaches could include microfluidic rupture tests or imaging modalities that detect early signs of clot deformation and vulnerability under shear. The diagnostic landscape could perhaps also benefit from mechanistically determined biomarker development. Could fracture toughness be estimated from surrogate plasma markers, such as crosslinked fibrin degradation products or the measurement of FXIII activity, FXIII A_2_B_2_, or FXIII-B levels? Could any of these markers or the combination of them help to identify patients at heightened embolic risk?

Undeniably, by showing that FXIIIa-mediated fibrin crosslinking enhances the fracture toughness of clots—independent of gross structural changes—the study by Ramanujam et al. [3] reframes how we think about thrombus rupture and embolization . Future research should aim to validate these findings *in vivo*, explore their applicability across diverse clinical contexts, and develop diagnostic tools and personalized management opportunities that reflect this new understanding of clot mechanics. In doing so, we move closer to a precision approach for assessing and mitigating embolic risk.
